# The physiological determinants of near-infrared spectroscopy-derived regional cerebral oxygenation in critically ill adults

**DOI:** 10.1186/s40635-019-0247-0

**Published:** 2019-05-02

**Authors:** Michael D. Wood, Jill A. Jacobson, David M. Maslove, John G. Muscedere, J. Gordon Boyd, The Cerebral Oxygenation and Neurological Outcomes Following Critical Illness (CONFOCAL) Research Group

**Affiliations:** 1 Centre for Neuroscience Studies, Queen’s University, 18 Stuart St, Botterell Hall, Kingston, ON Canada; 2 Department of Psychology, Queen’s University, 62 Arch Street, 318 Craine Hall, Kingston, ON Canada; 3 Department of Critical Care Medicine, Queen’s University, Rm 22.2.359 Davies 2, Kingston General Hospital, 76 Stuart St, Kingston, ON K7L 2V7 Canada; 4 Department of Medicine, Queen’s University, Rm 4.5.310 Watkins C, Kingston General Hospital, 76 Stuart St, Kingston, ON Canada

**Keywords:** Near-infrared spectroscopy, Critical illness, Brain tissue oxygenation, Cerebral oximetry, Cerebral perfusion

## Abstract

**Background:**

To maintain adequate oxygen delivery to tissue, resuscitation of critically ill patients is guided by assessing surrogate markers of perfusion. As there is no direct indicator of cerebral perfusion used in routine critical care, identifying an accurate strategy to monitor brain perfusion is paramount. Near-infrared spectroscopy (NIRS) is a non-invasive technique to quantify regional cerebral oxygenation (rSO_2_) that has been used for decades during cardiac surgery which has led to targeted algorithms to optimize rSO_2_ being developed. However, these targeted algorithms do not exist during critical care, as the physiological determinants of rSO_2_ during critical illness remain poorly understood.

**Materials and methods:**

This prospective observational study was an exploratory analysis of a nested cohort of patients within the CONFOCAL study (NCT02344043) who received high-fidelity vital sign monitoring. Adult patients (≥ 18 years) admitted < 24 h to a medical/surgical intensive care unit were eligible if they had shock and/or required mechanical ventilation. Patients underwent rSO_2_ monitoring with the FORESIGHT oximeter for 24 h, vital signs were concurrently recorded, and clinically ordered arterial blood gas samples and hemoglobin concentration were also documented. Simultaneous multiple linear regression was performed using all available predictors, followed by model selection using the corrected Akaike information criterion (AICc).

**Results:**

Our simultaneous multivariate model included age, heart rate, arterial oxygen saturation, mean arterial pressure, pH, partial pressure of oxygen, partial pressure of carbon dioxide (PaCO_2_), and hemoglobin concentration. This model accounted for a significant proportion of variance in rSO_2_ (*R*^2^ = 0.58, *p* < 0.01) and was significantly associated with PaCO_2_ (*p* < 0.05) and hemoglobin concentration (*p* < 0.01). Our selected regression model using AICc accounted for a significant proportion of variance in rSO_2_ (*R*^2^ = 0.54, *p* < 0.01) and was significantly related to age (*p* < 0.05), PaCO_2_ (*p <* 0*.*01), hemoglobin (*p* < 0.01), and heart rate (*p* < 0.05).

**Conclusions:**

Known and established physiological determinants of oxygen delivery accounted for a significant proportion of the rSO_2_ signal, which provides evidence that NIRS is a viable modality to assess cerebral oxygenation in critically ill adults. Further elucidation of the determinants of rSO_2_ has the potential to develop a NIRS-guided resuscitation algorithm during critical illness.

**Trial registration:**

This trial is registered on clinicaltrials.gov (Identifier: NCT02344043), retrospectively registered January 8, 2015.

**Electronic supplementary material:**

The online version of this article (10.1186/s40635-019-0247-0) contains supplementary material, which is available to authorized users.

## Background

The resuscitation phase (i.e., first 24–48 h) of critical illness is directed at maintaining adequate oxygen delivery to tissues to end-organ injury. Several organ systems have objective surrogate markers that can be serially monitored to ensure optimal end-organ perfusion. For example, renal perfusion is monitored by serially measuring urine output and serum creatinine levels [1]. In contrast, there is currently no well-defined proxy of cerebral perfusion used in routine clinical practice. This constitutes an important gap in our understanding of critical illness, as neuronal ischemia is a universal pathological finding in patients who die in the intensive care unit (ICU) [2]. In the absence of objective, quantitative markers, clinicians in the ICU rely upon the neurological exam (e.g., evaluating alertness, orientation, and ability to follow motor commands) [3] to assess brain perfusion. This clinical exam is often confounded by sedation, analgesia, and severity of illness. Therefore, clinical assessments may be unreliable in this setting and an alternative strategy to accurately monitor cerebral perfusion is needed to prevent irreversible neuronal injury.

Near-infrared spectroscopy (NIRS) is a simple and non-invasive technique to quantify regional cerebral oxygenation (rSO_2_). An adhesive sensor and light source are placed on the forehead, which emit varying wavelengths of infrared light (e.g., 700–1000 nm) that pass through the skin and bone with minimal absorption to an approximate depth of 2–3 cm of cerebral tissue [4]. The light that returns to the sensor represents the amount of spectral absorption occurring in the tissue bed (i.e., changes in oxygenated- and deoxygenated-hemoglobin), with venous circulation accounting for the majority of the signal (75–80%) [5], and is typically displayed as an absolute value ranging from 0 to 99%. The NIRS signal correlates with other measures of brain perfusion (e.g., jugular venous bulb oxygen saturation [6], brain tissue oxygen tension [7], and CT perfusion [8]), and its feasibility in critical care research has already been demonstrated [9].

Furthermore, NIRS has been described for many years in the cardiac anesthesiology literature. A targeted algorithm to optimize rSO_2_ during cardiac surgery has been developed [10, 11], which offers the potential to detect changes in cerebral perfusion and guide clinician intervention, and it has been demonstrated that nearly every episode (i.e., 97%) of cerebral desaturation can be successfully reversed [12]. However, outside of the operating room, the physiological determinants of cerebral oxygenation are poorly understood. As a result, targeted algorithms do not currently exist to optimize rSO_2_ in the ICU. This may be relevant, as we have recently demonstrated that low rSO_2_ is an independent risk factor for the subsequent development of delirium in critically ill patients [13]. A nested cohort within that prospective observational study underwent high-fidelity vital sign monitoring. The objective of the present study was to define the hemodynamic and physiological determinants of the NIRS-derived rSO_2_ from that nested cohort of critically ill patients.

## Material and methods

### Study design and recruitment

The Cerebral Oxygenation and Neurological outcomes FOllowing CriticAL illness (CONFOCAL) study (NCT02344043 clinicaltrials.gov) was a single-center prospective observational study (*n* = 103) for which the full protocol has been previously published [14]. As our protocol advanced from feasibility to a single-center pilot study, so did our data collection strategy, which initially began as hourly recordings documented in the electronic health records and advanced to continuous vital sign monitoring described below. The current manuscript is an exploratory analysis of a nested cohort of patients (*n* = 43) who received high-fidelity vital signs monitoring throughout the CONFOCAL study. Briefly, adult patients (≥ 18 years) admitted < 24 h to a 33-bed general medical/surgical and trauma ICU were eligible if they required mechanical ventilation with an expected duration > 24 h and/or having shock of any etiology. Shock was defined by vasopressor requirement at pre-specified doses [14]. Participants were excluded if they had a life expectancy < 24 h, a pre-ICU diagnosis of cognitive dysfunction as indicated by their medical records, or a primary central nervous system diagnosis (e.g., traumatic brain injury).

### Data acquisition: rSO_2_, vital sign monitoring, and blood gas collection

Immediately following enrolment, patients underwent rSO_2_ monitoring with the FORESIGHT monitor (CASMED, Caster Medical, Canada). For the majority of patients, a single 5-cm sensor was placed on the center of the patients’ forehead, > 3 cm from the superior rim of the orbit to avoid the frontal sinus [15], and recorded for 24 h. As the more traditional sensor placement used for patients undergoing cardiac surgery is bilaterally on the frontal lobes [16], a subset of CONFOCAL patients (*n* = 10) received an additional bilateral sensor (right) to quantify the level of agreement between the two NIRS sensors. This analysis indicated that the sensors shared an acceptable level of agreement (see Additional file 1: Figure S1) and that one sensor was adequate. These rSO_2_ recordings were not revealed to the treating clinicians. To assess the relationships among patient hemodynamics with the rSO_2_ recordings, we used commercially available software (Bedmaster, Excel Medical Electronics, FL, USA) to simultaneously capture the following high-frequency vital signs: heart rate (HR), arterial oxygen saturation (SpO_2_), systolic and diastolic blood pressure, and mean arterial pressure (MAP). These data were captured locally and stored on dedicated servers at the Queen’s University Centre for Advanced Computing (www.cac.queensu.ca). We also documented arterial and central venous blood gases, as well as hemoglobin concentration (Hb), when ordered clinically throughout this 24-h period of recording. As the first 24–48 h of critical care are guided at resuscitation of the patient, we chose to record for the first 24 h a patient’s ICU stay as understanding the determinants of rSO_2_ during this crucial period are foundational to the future development of targeted algorithms to optimize cerebral oxygenation.

### Data cleaning: detecting and editing data abnormalities

As databases containing high-frequency vital sign recordings are known to contain artifacts [17], we undertook data validation and cleaning steps to minimize the inclusion of these in our analysis. We removed missing data, as well as outliers based on cutoffs determined by inspection of histograms, see Additional file 2: Figure S2. Specifically, we removed HR values < 44 or > 134, MAP > 130 or < 39, SpO_2_ < 80, and rSO_2_ < 50 or > 85. We also removed data that were logically inconsistent, such as measures where the diastolic pressure was higher than the systolic or when values equaled 0.

### Data analysis

#### Determinants of the NIRS-derived rSO_2_ signal

All statistical analyses were performed using the R software version 3.3.2 [18]. Due to the variability of data collection (i.e., high frequency vs clinically ordered) presented above, *all* physiological variables were condensed to a 24-h mean prior to regression analysis in order to ensure that the data included were independent values rather than repeated recordings, which would violate the independence assumption of linear regression. Our primary objective was to assess the hemodynamic and physiological variables (i.e., determinants of oxygen delivery) predicting rSO_2_ during the first 24 h of critical illness. Therefore, simultaneous multiple linear regression was performed using HR, SpO_2_, MAP, arterial pH, arterial partial pressure of oxygen (PaO_2_), arterial partial pressure of carbon dioxide (PaCO_2_), and Hb as predictors. Although systolic and diastolic blood pressures were recorded, we only included MAP due to redundancy. As increasing age has been associated with decreases in cerebral blood flow (CBF) [19], and potentially rSO_2_, we included age as a covariate. A correlation matrix illustrating the various relationships between the predictors of rSO_2_ can be observed in Additional file 3: Figure S3. Due to potential overfitting, model selection was implemented to generate a reduced multivariate regression model using the MuMIn package for R [20]. This package was used to iteratively compare all possible models given the data, whereas other model selection techniques may drop predictors in a stepwise fashion (i.e., backwards or forwards) [21] and only evaluate a small fraction of all possible subsets of the data. Due to our relatively small sample size potentially biasing our analysis, we applied the corrected AIC method (AIC_c_) [22] instead of implementing the Akaike’s information criterion (AIC) [23] as the model criterion. The lowest AIC_c_ value represents the most parsimonious model accounting for a large amount of variance with as few predictors as possible, so as not to over- or under-fit the model while minimizing information loss. Diagnostic testing indicated that the residuals were normally distributed, had equal variances, and did not suggest substantial evidence of collinearity among predictors (data not shown).^1^ The regression model and individual predictors were considered statistically significant if *p* < 0.05. The simultaneous and the selected regression model were compared using AIC.

#### Sensitivity power analysis

We performed a sensitivity power analysis to determine the minimum effect size that our regression analysis, based on model selection using AICc, could have detected given the data. We used the following model parameters: sample size = 43, power = 0.90, number of predictors = 4, and *α* = 0.05, which indicated that we could have detected a minimum effect of *R*^2^ = 0.289. The sensitivity power analysis was performed using G*Power [24] version 3.1.9.2.

## Results

### Patient characteristics

**Fig. 1:**
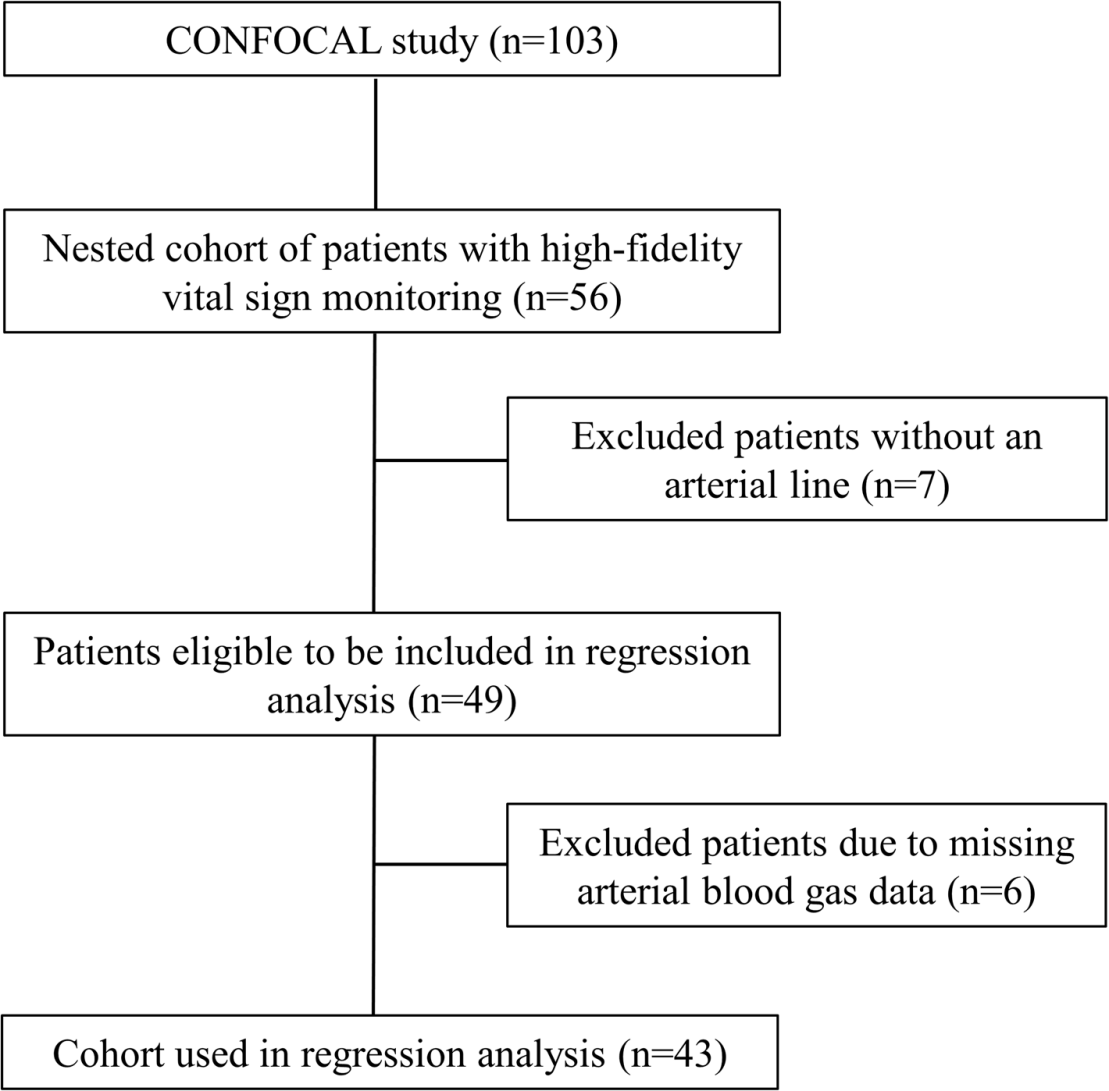
CONSORT diagram demonstrating patient inclusion and exclusion during patient recruitment and subsequent data analysis

From March 2014 to September 2016, 1155 patients were assessed for eligibility and 104 were enrolled. Of this cohort (*n* = 103), 56 patients (54%) underwent high-frequency vital sign monitoring from August 2015 to September 2016, 7 of which (13%) were excluded because they did not have an arterial line present. Of the remaining 49 patients, 6 were excluded due to missing or insufficient arterial blood gas data, resulting in the inclusion of 43 subjects in the regression analysis (Fig. 1). The primary admitting diagnoses were mainly respiratory failure (30%), followed by severe sepsis/septic shock (16%), cardiac (16%), and gastrointestinal (16%), whereas previous comorbidities largely included a history of hypertension (56%), cardiac complications (44%), and respiratory disease (33%). Most patients were intubated at the time of enrollment (95%), and approximately half (56%) were being treated with vasoactive agents. The median age was 68 years (IQR, 58.5–79), most patients were male (67%), median length of ICU stay was 7 days (IQR, 4 to 13), and ICU mortality was 7 (16%). Full demographics and clinical characteristics are shown in Table 1.

**Table 1:** Demographics and clinical characteristics

Characteristic	Nested cohort (*n* = 43)
Median age (years, [IQR])	68 [58.5–79]
Male gender (no. [%])	29 [67]
Admitting diagnosis (no. [%]):	
Respiratory failure	13 [30]
Severe sepsis/septic shock	7 [16]
Cardiac	7 [16]
Gastrointestinal	7 [16]
Vascular	4 [9]
Trauma	3 [7]
Neurological	0
Other*	2 [5]
APACHE score (median, IQR)	20 [16–26]
Co-morbidities (no. [%]):	
Cardiac**	19 [44]
Hypertension	24 [56]
Respiratory***	14 [33]
Diabetes	11 [26]
Active tobacco use	11 [26]
Heavy alcohol use	5 [12]
At time of enrolment (no. [%]):	
Intubated	41 [95]
Vasoactive agents	24 [56]
ICU LOS (median [IQR])	7 [4–13]
ICU mortality (no. [%])	7 [16]
Physiological variables (median [IQR]):	
MAP (mmHg)	73.77 [71.04–80.84]
HR (bpm)	85.77 [73.82–99.79]
PaCO_2_ (mmHg)	40.50 [35.20–44.75]
PaO_2_ (mmHg)	86.25 [79.75–94.17]
pH (mmHg)	7.37 [7.33–7.42]
SpO_2_ (%)	96.34 [95.23–97.52]
Hb (g/L)	102.67 [86.00–115.50]
rSO_2_ (%)	68.41 [64.56–72.16]

*MAP* mean arterial pressure, *HR* heart rate, *PaCO*_*2*_ arterial partial pressure of carbon dioxide, *PaO*_*2*_ arterial partial pressure of oxygen, *SpO*_*2*_ arterial oxygen saturation, *Hb* hemoglobin, *rSO*_*2*_ regional cerebral oxygenation

*Other included the following: drug overdose/withdrawal and acute kidney injury

**Cardiac included the following: arrhythmia, prior myocardial infarction, prior cardiac arrest, known coronary artery disease, and/or congestive heart failure

***Respiratory included the following: asthma or COPD

### Data collection and analysis

High-frequency vital sign monitoring was recorded for a median duration of 23.95 h (IQR, 23.23–24.02), yielding more than 350,000 individual vital sign measurements, including rSO_2_ recordings. Nearly 20,000 data points were removed due to missing data (5.5%), and a further 79 removed due to zero or negative values which were deemed erroneous. We inspected histograms of the distribution of each vital sign to identify cutoffs distinguishing true physiologic measures from likely artifacts. Based on these cutoffs, a further 6866 observations (2%) were removed (see Additional file 4: Figure S4). Compared to the low amount of missing vital sign data, the physiological data obtained from clinically ordered central venous blood gases data were sparse and subsequently excluded from our analysis due to a high degree of missingness. For example, all 43 patients had at least 1 arterial blood gas recording, where 28 patients did not have a venous blood gas sample during the first 24 h of their ICU stay. Furthermore, the median collected arterial blood gas samples was 2 (IQR, 1–3), whereas the median collected venous blood gas samples was 0 (IQR, 0–1). Median Hb collection throughout the 24 h of recording was 1 (IQR, 1–2). Therefore, most of the available data was successfully captured for the vital sign monitoring and arterial blood gas data, but a substantial amount of central venous blood gas data was missing or inadequate for subsequent regression analysis.

### Physiological determinants of rSO_2_ during critical illness

#### Simultaneous multiple regression analysis

Our simultaneous regression model included all available variables that could influence cerebral oxygen delivery (i.e., age, HR, SpO_2_, MAP, pH, PaO_2_, PaCO_2_, and Hb concentration). This model accounted for a significant proportion of variance in the NIRS-derived rSO_2_ signal, *R*^2^ = 0.58, *F* (8, 34) = 5.845, *p* < 0.01. However, PaCO_2_ and Hb concentrations were the only significant predictors in the regression model, *b* = 0.165, *t*(34) = 2.035, *p* < 0.05, and *b* = 0.086, *t*(34) = 2.772, *p* < 0.01, respectively. Both predictors had a positive relationship with rSO_2_ during the first 24 h of critical illness. However, several predictors were included in this model, which may have decreased precision of the regression coefficients. This is evidenced by the substantial difference observed between the overall *R*^2^ and the adjusted *R*^2^ of 0.48. The full regression results are shown in Table 2.

**Table 2:** Regression models predicting the near-infrared spectroscopy regional cerebral oxygenation signal in critically ill adult patients

Predictor	Simultaneous regression model			AICc selected regression model		
	*b*	*β*	*r*	*b*	*β*	*r*
Age	− 0.08 [− 0.17, 0.01]	− 0.22 [− 0.48, 0.03]	− 0.32*	− 0.10 [− 0.19, − 0.01]*	− 0.27 [− 0.51, − 0.04]	− 0.32*
HR	0.06 [− 0.02, 0.14]	0.19 [− 0.06, 0.44]	0.22	0.08 [0.01, 0.15]*	0.25 [0.02, 0.49]	0.22
SpO_2_	− 0.37 [− 1.19, 0.44]	− 0.15 [− 0.48, 0.18]	− 0.42**			
MAP	0.02 [− 0.16, 0.20]	0.03 [− 0.23, 0.28]	0.22			
pH	− 16.44 [− 38.52, 5.65]	− 0.19 [− 0.45, 0.07]	− 0.34*			
PaO_2_	0.05 [− 0.05, 0.15]	0.13 [− 0.14, 0.41]	− 0.07			
PaCO_2_	0.16 [0.00, 0.33]*	0.30 [0.00, 0.59]	0.57**	0.21 [0.07, 0.34]**	0.37 [0.13, 0.62]	0.57**
Hb	0.09 [0.02, 0.15]**	0.40 [0.11, 0.69]	0.43**	0.09 [0.04, 0.14]**	0.41 [0.16, 0.66]	0.43**
Fit	*R*^2^ = 0.579** [0.22, 0.65]; adjusted *R*^2^ = 0.480			*R*^2^ = 0.535 [0.25, 0.65]**; adjusted *R*^2^ = 0.487		

*b* represents unstandardized regression weights. *β* indicates the standardized regression weights. *r* represents the zero-order correlation. The [] indicate the lower and upper limits of the 95% confidence interval, respectively. The gray-shaded region represents the predictors that were not retained following model selection

*AICc* corrected Akaike information criterion, *HR* heart rate, *SpO*_*2*_ arterial oxygen saturation, *MAP* mean arterial pressure, *PaO*_*2*_ arterial partial pressure of oxygen, *PaCO*_*2*_ arterial partial pressure of carbon dioxide, *Hb* hemoglobin

*p < 0.05

**p < 0.01

#### Model selection using AICc and multiple regression

**Fig. 2:**
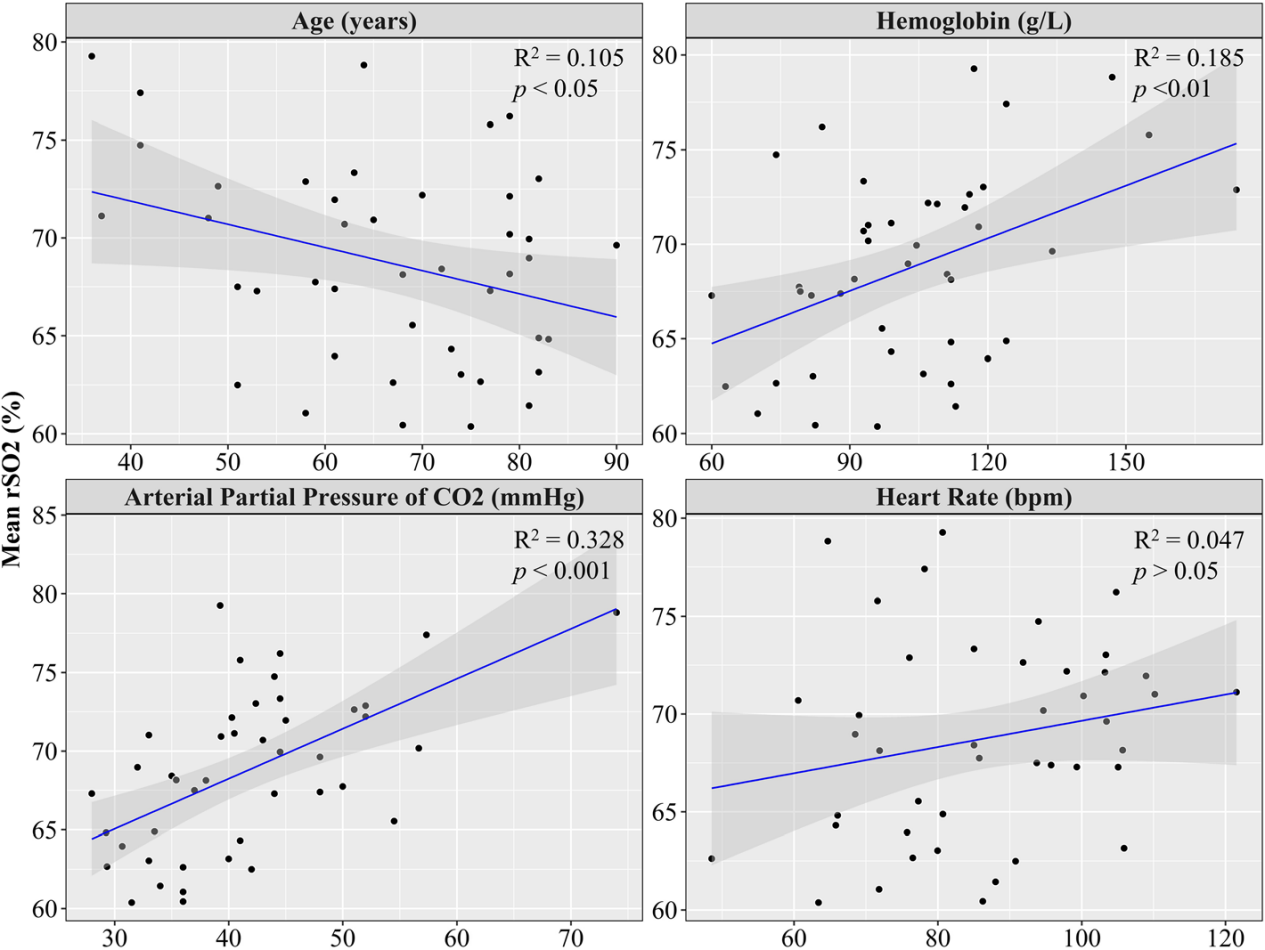
Scatter plots illustrating the various relationships between regional mean cerebral oxygenation (rSO_2_) recordings and mean levels of various predictors of oxygen delivery (i.e., age, hemoglobin, partial pressure of carbon dioxide, and heart rate). Black data points represent each individual patient with the blue line representing a linear model fit to the data and the gray-shaded region representing the 95% confidence interval

Model selection based on the AIC_c_ indicated that the top model (i.e., lowest AIC_c_) included the following predictors: age, PaCO_2_, Hb, and HR. Regression analysis of this selected model accounted for a significant proportion of variance in the rSO_2_ signal, *R*^2^ = 0.536, *F* (4, 38) = 10.95, *p <* 0*.*01, and a comparable adjusted *R*^2^ of 0.49 was observed for this reduced model. Furthermore, this analysis indicated that the percentage of rSO_2_ increased significantly with increases in PaCO_2_, *b =* 0.208, *t*(38) *=* 3.062, *p* < 0.01; Hb concentration, *b* = 0.089, *t*(38) = 3.357, *p* < 0.01; and HR, *b* = 0.079, *t*(38) = 2.230, *p* < 0.05 (Table 2 ). In contrast, the percentage of rSO_2_ significantly decreased as age increased, *b* = − 0.100, *t*(38) = − 2.329, *p* < 0.05. The unadjusted effects of the significant predictors on rSO_2_ can be observed in Fig. 2. Model comparison using AIC indicated that the selected regression model (i.e., age, PaCO_2_, Hb, and HR) had a lower AIC when compared to the simultaneous regression model (AIC = 239.64 and 243.41, respectively), which is further evidenced by the minor difference in variance accounted for by each model, but the selected model includes substantially fewer predictors.

## Discussion

It has been argued that intraoperative NIRS monitoring should be the standard of care [25], as NIRS has already demonstrated clinically relevant results when used to monitor rSO_2_ throughout cardiac surgery including fewer incidences of organ dysfunction and shorter ICU stays [26, 27]. However, it is unknown if the determinants of cerebral perfusion during cardiac surgery are similar among critically ill patients who may have dissimilar physiological derangements as part of their illness. We used multivariate modeling to assess the physiological determinants of the NIRS-derived rSO_2_ signal during the first 24 h of critical illness in adult patients. Our selected regression model suggested that PaCO_2_, HR, and Hb concentration may be possible therapeutic targets to optimize cerebral oxygenation, as our model accounted for > 50% of the variance in rSO_2_. As this analysis was exploratory, we conducted a sensitivity power analysis. Given that we were powered to detect an effect approximately half of what we observed, there is improved confidence that our analysis was not biased for only detecting inflated effect sizes [28]. As this study was based on a single center, our results will need to be further validated in a larger and more diverse cohort of critically ill patients.

### Relationship between known determinants of oxygen delivery and rSO_2_

As a preliminary step towards demonstrating the utility of NIRS as a surrogate marker of cerebral perfusion in critically ill patients, we assessed the relationship between rSO_2_ and other variables related to oxygen delivery that are monitored in routine clinical practice. It is well accepted that the amount of oxygen delivered to tissues is the product of arterial oxygen content and cardiac output. Arterial oxygen content is determined by Hb concentration, SpO_2_, and oxygen dissolved in blood (PaO_2_ × 0.003 mL/mmHg O_2_/dL blood) [29], whereas cardiac output is determined by HR and stroke volume [30]. Of these determinants of oxygen delivery, we were able to measure Hb, HR, SpO_2_, and PaO_2_.

Red blood cell (RBC) transfusion to improve tissue oxygen delivery is a common ICU intervention administered to approximately 1/3 of critically ill patients [31]. However, this practice still remains controversial due to the potential complications of transfusion (e.g., transfusion-related acute lung injury, altered coagulation, or infections) [32], as well as studies indicating that the restrictive vs liberal use of RBC transfusions may result in similar rates of mortality [33–35] and ischemic events [35]. Importantly, neurological outcomes have not been assessed in these large randomized trials of transfusion thresholds. This issue is further complicated by a lack of understanding of the neurophysiologic effects of RBC transfusion on cerebral oxygenation. In patients with severe traumatic brain injury, mean pre- and post-transfusion Hb concentrations were significantly different, but this significant difference was not observed for pre- and post-rSO_2_ recordings [36]. This may have been due to the small sample size and potentially underpowered (*n* = 19) analyses. Our study on critically ill, non-brain-injured patients demonstrated a significant positive association between Hb concentration and rSO_2_. This inconsistency between studies may be partially explained by different patient populations being assessed, as patients with traumatic brain injury may have structural etiologies that interfere with the NIRS signal, such as cerebral contusions or hematomas. Our finding raises the possibility that increasing Hb concentrations within a therapeutic window may be a component of developing a clinical algorithm to optimize rSO_2_ during critical illness. However, the effects of RBC transfusions on rSO_2_, as well as subsequent clinical outcomes, need further analysis.

With regard to cardiac output, we demonstrated a significant positive association between HR and rSO_2_. This might suggest that medications that increase heart rate (e.g., dobutamine) could be part of an algorithm to increase cerebral oxygenation. However, it is important to acknowledge that our study did not have data on stroke volume to directly calculate cardiac output. Future studies may wish to utilize non-invasive assessments of cardiac output in order to directly assess the association between cardiac output and rSO_2_.

Clinicians routinely depend on data derived from pulse oximetry to monitor tissue oxygen saturation in critically ill patients. However, these recordings only provide information regarding the arterial blood content from peripheral tissues. In our study, there was a negative association between rSO_2_ and SpO_2_. Furthermore, SpO_2_ was not identified as a significant predictor of rSO_2_ when controlling for the other determinants of oxygen delivery. This may suggest that pulse oximetry may inadequately assess cerebral oxygenation, which further argues for the need for routine monitoring of rSO_2_ at the bedside. Alternatively, this non-significant finding may simply reflect the restricted range of SpO_2_ levels collected among patients, as arterial oxygen saturation is tightly regulated within the ICU. Due to the small sample size, our analysis may have been underpowered to detect this effect in a multivariate regression model and further analysis is warranted. Furthermore, as mentioned previously, the NIRS signal is mostly comprised of venous oxygenation [5]. Therefore, rSO_2_ may be more related to intracranial venous oxygen saturation, partially explaining the negative association observed with SpO_2_, and may reflect the interplay between oxygen delivery and consumption. Although rSO_2_ has been shown to be positively correlated with central venous oxygen saturation previously [37], we had an insufficient sample of central venous oxygen saturation data to assess this relationship in our regression analysis.

In the present study, we did not find a significant association between rSO_2_ and PaO_2_. Since the oxygen-hemoglobin dissociation curve becomes relatively flat when oxygen saturation is > 90% and PaO_2_ is above 80 mmHg (i.e., sigmoid shape), increases in PaO_2_ have relatively little impact on saturation/content (i.e., inhaled oxygen will increase PaO_2_ levels but there will only be a minimal increase in blood oxygen content) [38]. Our cohort had median SpO_2_ and PaO_2_ values well above these values mentioned previously, which may partially explain the observed non-significant association. However, had a larger range of values been collected for PaO_2_ and SpO_2_, it stands to reason that a significant association(s) may have been observed with rSO_2_ due to the substantial dependence of oxygen content on PaO_2_, which would have also been reflected by subsequent changes in SpO_2_ levels.

### PaCO_2_ may be directly associated with rSO_2_ in critically ill patients

Respiratory gases, such as PaCO_2_, have substantial effects on the radius of cerebral blood vessels (e.g., increases in PaCO_2_ cause cerebral vasodilation, thus increasing CBF) [39]. However, metabolic acidosis is frequently compensated by spontaneous or controlled hyperventilation during the resuscitation of critically ill patients. As cerebral perfusion is not routinely monitored at the bedside in critically ill patients, the effects of hyperventilation, and subsequent hypocapnia, remain unclear. Since we found a significant positive association between PaCO_2_ and rSO_2_, prolonged hyperventilation during critical illness may result in the unintended consequence of compromised cerebral perfusion and potentially secondary neuronal injury.

### Physiological parameters NOT associated with rSO_2_: PaO_2_ and MAP

We describe above the possible explanation(s) for the non-significant association between rSO_2_ and PaO_2_. A similar nonsignificant association between MAP and rSO_2_ was also observed, which may be related to intact cerebral autoregulation as CBF is preserved through a range of MAP values (i.e., 50–150 mmHg) [40, 41]. A limitation of the current study is that the integrity of cerebral autoregulation is not captured in this cohort.

### Limitations and future directions

Analyzing the physiological determinants of rSO_2_ in our cohort of patients was limited by our single-center design and by the small number of patients that underwent high-frequency vital sign recording. Therefore, our findings will need to be validated among a larger cohort of critically ill patients. Additional measures of tissue oxygenation (e.g., SjvO_2_, lactate) would ideally be incorporated into the regression analysis. However, these lab tests were sent infrequently during the 24-h period of NIRS recording such that the data could not be used. Furthermore, quantifying patient cardiac output, rather than recording only heart rate, may provide important information regarding the NIRS association with determinants of oxygen delivery. As stated previously, the NIRS signal is primarily derived from venous circulation (75–80%) [5]. Our analysis included only arterial blood gas data, which may partially explain why ~ 50% of the variance of the NIRS signal was not accounted for by our regression model. Furthermore, given our small sample size, we could not adjust for other potentially relevant covariates that might influence cerebral oxygenation, such as admitting diagnosis, medications, or medical history. Additionally, due to the heterogeneity at which our data was collected (e.g., continuous recording vs clinically ordered), we reduced our data set to a 24-h mean per patient. Future analyses may want to systematically collect all physiological data and conduct a mixed effects regression analysis to account for repeated measurements and potential individual variability among ICU patients. Furthermore, the right and center sensors largely agreed throughout the recording period, which suggests that one sensor in the middle of the forehead may be adequate. However, further research is needed to investigate if the center placement detects focal desaturations that may be clinically meaningful (e.g., right-sided stroke) and better detected using traditional two bilateral sensors, which will be imperative to developing a target algorithm to optimize rSO_2_. Lastly, age had a significant negative association with rSO_2_ and will need to be included in future analyses to adjust for this age-related decrease in cerebral perfusion. However, the exact age-related mechanism(s) associated with this decrease in rSO_2_ is unclear. Despite these limitations, however, we identified three predictors (i.e., PaCO_2_, HR, Hb) of the NIRS-derived rSO_2_ signal that are clinically established determinants of oxygen delivery, which provide evidence that NIRS may be a suitable marker for monitoring cerebral oxygenation during critical illness.

## Conclusions

Our analysis provides evidence that the NIRS-derived rSO_2_ signal is a viable neuromonitoring modality to assess cerebral oxygenation during critical illness, as this signal was predicted by known and reliable clinical determinants of oxygen delivery during the first 24 h of critical illness. Further elucidation of the determinants of the rSO_2_ signal may be useful in developing resuscitation algorithms designed to optimize cerebral oxygenation, an important therapeutic target among critically ill patients. However, clinically relevant covariates will need to be modeled in future analyses.

## Additional files

Additional file 1: **Figure S1.** Repeated measures Bland Altman plot indicating that the pooled data across the center and right sensors display a high level of agreement. *Note*. The red dotted lines indicate the 95% limits of agreement (i.e., the two sensors mean minus 1.96 SD and plus 1.96 SD). The black line represents the mean (i.e., bias) of recordings across sensors. Black dots represent pooled recordings of regional cerebral oxygenation across 10 intensive care unit patients. As an illustrative example, the Bland Altman analysis indicated that the mean difference (i.e., bias) between the sensors was − 0.31, which indicates that the right sensor on average records 0.31% higher than the center sensor. Furthermore, the lower and upper limits of agreement (− 7.45% and 6.84%, respectively) were minor, indicating that the sensors display high agreement. (JPEG 2129 kb)

Additional file 2: **Figure S2.** Histograms of high-frequency hemodynamic variable recordings used to remove anomalous data before conducting regression analysis. Diastolic BP = diastolic blood pressure; MAP = mean arterial pressure; systolic BP = systolic blood pressure; rSO_2_ = regional cerebral oxygenation; SpO_2_ = arterial oxygen saturation. (PDF 37 kb)

Additional file 3: **Figure S3.** Matrix of the pooled correlation analysis of all predictors of regional cerebral oxygenation included in the simultaneous regression model. This plot provides visual representation of the associations between various hemodynamic/physiological parameters. The direction of the association is represented by the color (blue = positive; red = negative), and the strength is indicated by shading (dark = strong; light = weak). Each colored square has corresponding text, which represents the *p* value for the correlation analysis between the column and row parameter. The asterisks indicate significant Pearson correlations coefficients (*p* < 0.05). (JPG 7206 kb)

Additional file 4: **Figure S4.** Bar graph illustrating that amount of missing values per high-frequency vital sign recording. Diastolic BP = diastolic blood pressure; MAP = mean arterial pressure; systolic BP = systolic blood pressure; rSO_2_ = regional cerebral oxygenation; SpO_2_ = arterial oxygen saturation. (PDF 22 kb)

## Abbreviations

95% CI: 95% confidence interval
AIC: Akaike information criterion
AICc: Corrected Akaike information criterion
CBF: Cerebral blood flow
Hb: Hemoglobin concentration
HR: Heart rate
IQR: Interquartile range
MAP: Mean arterial pressure
NIRS: Near-infrared spectroscopy
PaCO_2_: Arterial partial pressure of carbon dioxide
PaO_2_: Arterial partial pressure of oxygen
RBC: Red blood cell
rSO_2_: Regional cerebral oxygenation
SjvO_2_: Jugular venous oxygen saturation
SpO_2_: Peripheral oxygen saturation

## Footnotes

1 Another assumption of regression is linearity for which only one of the predictors violated this assumption, SpO_2_. Therefore, we also tested a version of the simultaneous model that included the linear and quadratic terms for SpO_2_. In this model, both linear and quadratic terms for SpO_2_ were significant predictors of rSO_2_, *b* = 48.47, *t*(33) = 2.314, *p* = 0.027, *b* = − 0.26, *t*(33) = − 2.333, *p* = 0.026, respectively. Because the quadratic term would qualify the linear term, these results indicate that rSO_2_ is lower at moderate levels of SpO_2_ than at higher or lower levels of SpO_2_ arterial. However, complex terms like interactions and polynomials require larger sample sizes for stability. Consequently, we interpret these results with caution and await replication of this finding with a larger sample.
