# Supplementary figures and images for: The physiological determinants of near-infrared spectroscopy-derived regional cerebral oxygenation in critically ill adults
Source: Intensive Care Med Exp. 2019 May 2;7:23. doi: 10.1186/s40635-019-0247-0 (PMC6497723; doi:10.1186/s40635-019-0247-0)

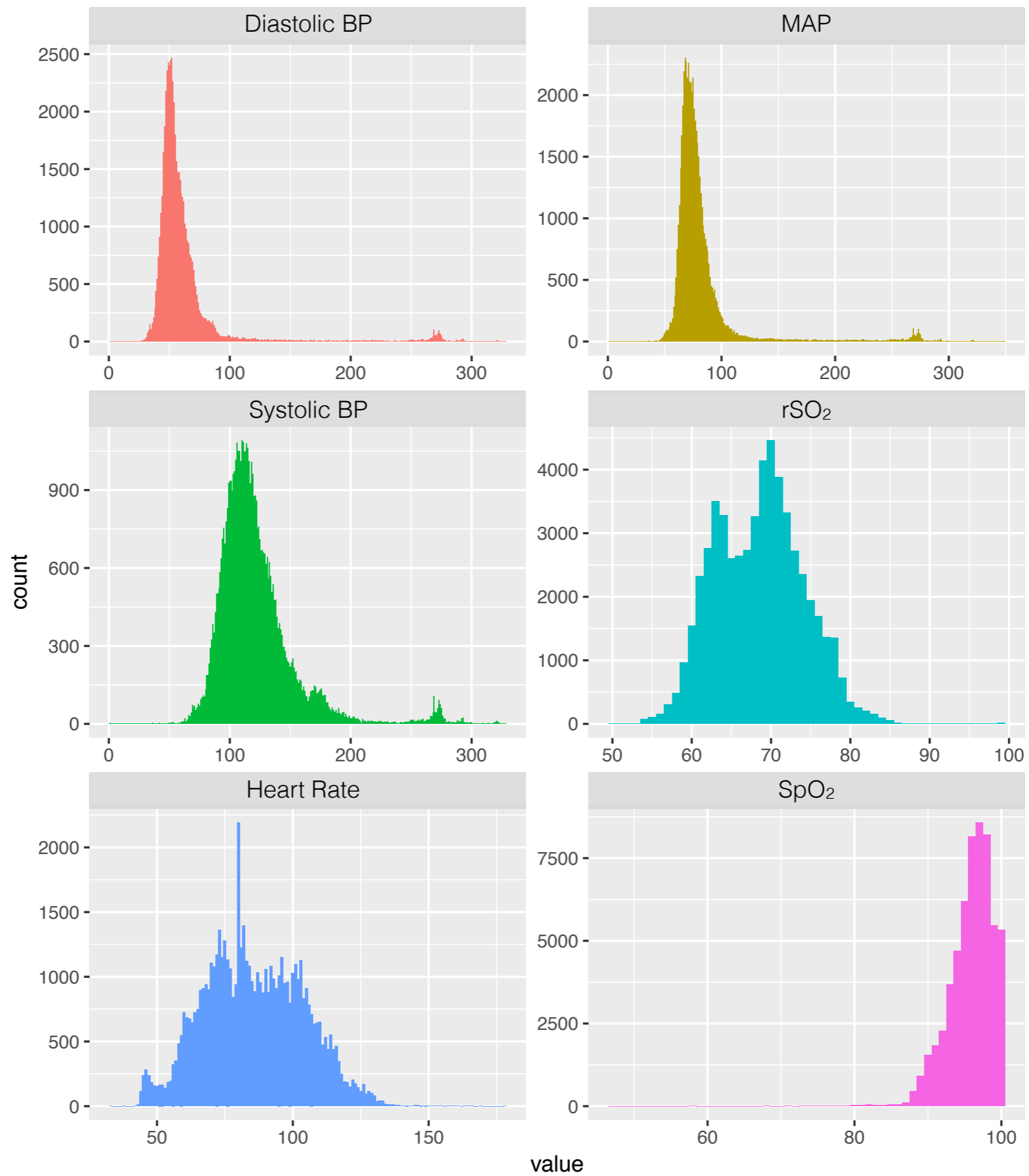

Supplement: Supplementary file 2 — Figure S2. Histograms of high-frequency hemodynamic variable recordings used to remove anomalous data before conducting regression analysis. Diastolic BP = diastolic blood pressure; MAP = mean arterial pressure; systolic BP = systolic blood pressure; rSO2 = regional cerebral oxygenation; SpO2 = arterial oxygen saturation. (PDF 37 kb) [file 40635_2019_247_MOESM2_ESM.pdf]

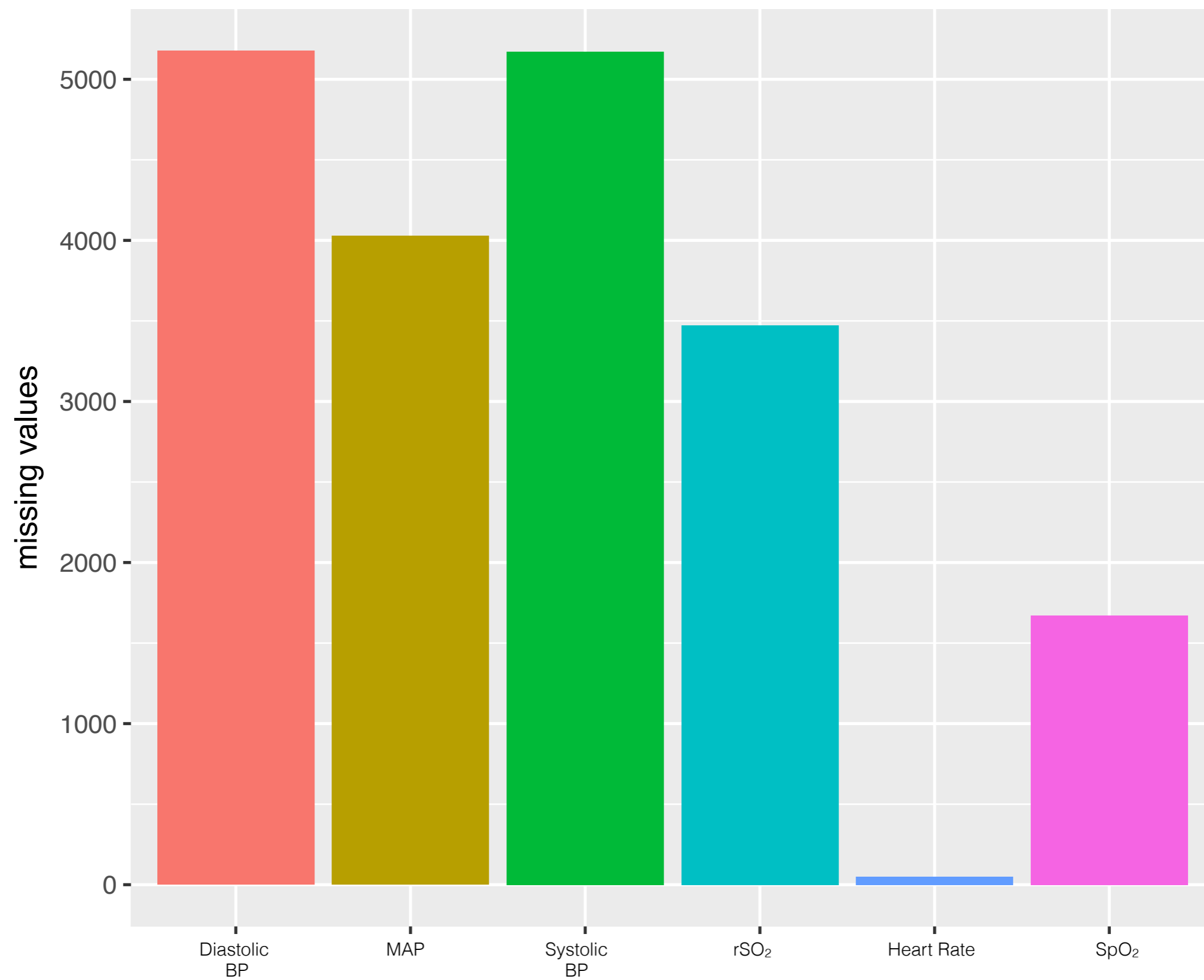

Supplement: Supplementary file 4 — Figure S4. Bar graph illustrating that amount of missing values per high-frequency vital sign recording. Diastolic BP = diastolic blood pressure; MAP = mean arterial pressure; systolic BP = systolic blood pressure; rSO2 = regional cerebral oxygenation; SpO2 = arterial oxygen saturation. (PDF 22 kb) [file 40635_2019_247_MOESM4_ESM.pdf]
